# Influence of suspended inorganic particles (kaolinite) on eggs and larvae of the pelagic shrimp *Lucensosergia lucens*

**DOI:** 10.1038/s41598-022-18373-8

**Published:** 2022-08-18

**Authors:** Md. Jahangir Alam, Kazuma Date, Hisayuki Arakawa

**Affiliations:** Tokyo University of Marine Science and Technology, 5-7, Konan-4, Minato, Tokyo, 108-8477 Japan

**Keywords:** Ecology, Ecology, Environmental sciences, Ocean sciences

## Abstract

The pelagic shrimp *Lucensosergia lucens* is a commercially important species in Japan, predominantly harvested in Suruga Bay. It has been suggested that a marked decrease in the wild population over recent years is associated with an increased concentration of suspended particles. We tested the hypothesis that suspended inorganic particles (kaolinite) negatively affect the hatching ratio of fertilized eggs, and the survival, growth, and metamorphosis of nauplius and elaphocaris larvae. The relative hatching ratio of eggs decreased from 100 to 57.7% at 139 mg L^−1^ of kaolinite particles. Similarly, the relative survival ratio of nauplius larvae progressively decreased from 100% in filtered seawater to 73.6% after 72 h of exposure to 139 mg L^−1^ of kaolinite particles. Consequently, the survival ratio of elaphocaris larvae was greatly reduced at high particle concentrations. Exponential growth in the standard lengths of elaphocaris larvae occurred at particle concentrations < 6.9 mg L^−1^, but growth was inhibited at kaolinite concentrations > 20 mg L^−1^.

## Introduction

The pelagic shrimp *Lucensosergia lucens*, known as ‘Sakura ebi’, is a commercially important species in Japan. It is a small shrimp, 4–5 cm long from jaw to tail when fully grown^1^. Its lifespan is approximately 15 months and it spawns once between late May and mid-November. The optimal temperature range for larval development is 18–25 °C^2^. The planktonic fertilized eggs hatch into nauplii, which metamorphose through elaphocaris, acanthosoma, mastigopus, and post-larval stages before maturing into adults^3^.

Natural populations of *L. lucens* occur in Suruga Bay and Sagami Bay, at the mouth of Tokyo Bay in Japan, and in the coastal waters of Tungkang along the east coast of Taiwan^2,4–7^. Large-scale fishing of *L. lucens* is carried out only in Suruga Bay, particularly in the coastal areas close to the Fuji River mouth.

Recently, catches of *L. lucens* have declined markedly, from an annual average of 2406 t in 2000 to 140 t in 2021 (http://www.pref.shizuoka.jp/j-no1/m_sakuraebi.html). Several factors have been suggested to be the cause of this depletion, including increased water temperature, increased predation pressure, and the meandering of the Kuroshio Current^8^. However, these factors seem inadequate to explain such a sharp decline in the population. The main spawning grounds for *L. lucens* are believed to be around the mouth of the Fuji River in inner Suruga Bay^2^. A recent increase in the turbidity of the Fuji River raises the possibility that suspended solids in seawater might also be contributing to the decline.

Turbidity in seawater is a result of suspended organic and inorganic matter^9^. Clay minerals, which are a major constituent of the inorganic portion of suspended particles in Suruga Bay are closely related to those of the surrounding land areas^10^. The major rivers draining into this bay have been reported to be the source of this relationship, and clay minerals may consist of kaolinite, chlorite, illite, and smectite^11–13^. Kaolinite is a typical particle that flows out of rivers, and most researchers analyzing the effect of inorganic particles on marine invertebrates prefer using kaolinite because of its inertness, thus avoiding the chemical effects associated with suspended inorganic materials^9,14–17^. Kaolinite is generally gray and white in color and has a density of 2.63 g/cm^3^^18^. The chemical formula for kaolinite is Al_4_Si_4_O_10_(OH)_8,_ and its components are SiO_2_ (46.54%), Al_2_O_3_ (39.50%), and H_2_O (13.96%)^19^. It has a low surface area and cation exchange capacity (< 1 centimole/kg), and does not swell in water^20^.

High concentrations of suspended particles in seawater are known to have negative effects on the behavior and physiology of marine organisms at various stages of their life cycles^21^. Lloyd^22^ reported that elevated levels of suspended sediments are lethal to juvenile salmonids, and that lower levels of suspended sediments could be associated with chronic sublethal effects, including reduced foraging capacity, decreased growth, lowered disease resistance, and enhanced stress. Elevated levels of suspended inorganic clay particles (illite, smectite, and kaolinite) have been associated with reduction in fecundity and brood size of *Daphnia ambigua*^23^ and cladocerans^24,25^, and delayed egg and larval development in oysters and clams^26^. Filter-feeding invertebrates are generally less tolerant to inorganic suspended particles than other aquatic species. High concentrations of clay particles, including kaolinite (50–100 mg L^−1^) lowered the ingestion rates of food in daphnids to starvation levels^27^, inhibited filtering rates in adult Manila clams *Ruditapes philippinarum*^17^, and impaired reproduction and the exchange rate of O_2_ in shrimp^28^, all of these effects result in the reduction of growth rates^26^ at a sublethal level, and ultimately mortality^29^. However, there are few reports of the influence of suspended particles on the early life stages of shrimp^29^.

Lin et al.^30^ observed that inorganic particle levels of 65 ± 15 NTU inhibited the osmoregulatory capacity of juvenile *Penaeus japonicus*, while a turbidity of 35 ± 15 NTU increased gill Na^+^–K^+^ ATPase activity. Suspended inorganic particles have also been observed to reduce immunological activity in *Penaeus vannamei*, indicated by reduced total hemocyte count, lower activities of phenoloxidase and superoxide dismutase, increased hemolymph osmolality, and elevated levels of stress metabolites, including glucose and lactate^28^. It has been pointed out that suspended inorganic particles can have a large effect on shrimp species, especially during their pelagic larval stages. The spawning season of *L. lucens* is long, and the total duration of the egg and larval stages is about 2 months^2^; we hypothesized that recent increases in the levels of suspended inorganic particles in seawater during the larval period of *L. lucens* might be adversely affecting its survival.

In this study, we tested the hypothesis that elevated levels of suspended inorganic (kaolinite) particles in seawater negatively affect the hatching ratio of fertilized eggs, and the survival, growth, and metamorphosis of nauplius and elaphocaris larvae of *L. lucens.*

## Results

### Hatching success

Fertilized eggs of *L. lucens* were put into seawater with different suspended particle (kaolinite) concentrations. After 38 h, most eggs had hatched and the number of larvae was counted. The mean hatching ratio in seawater without kaolinite was 94.7 ± 1.6%. This value was set at a relative hatching ratio of 100% for comparison with the effect of particle concentration on hatching.

**Figure 1:**
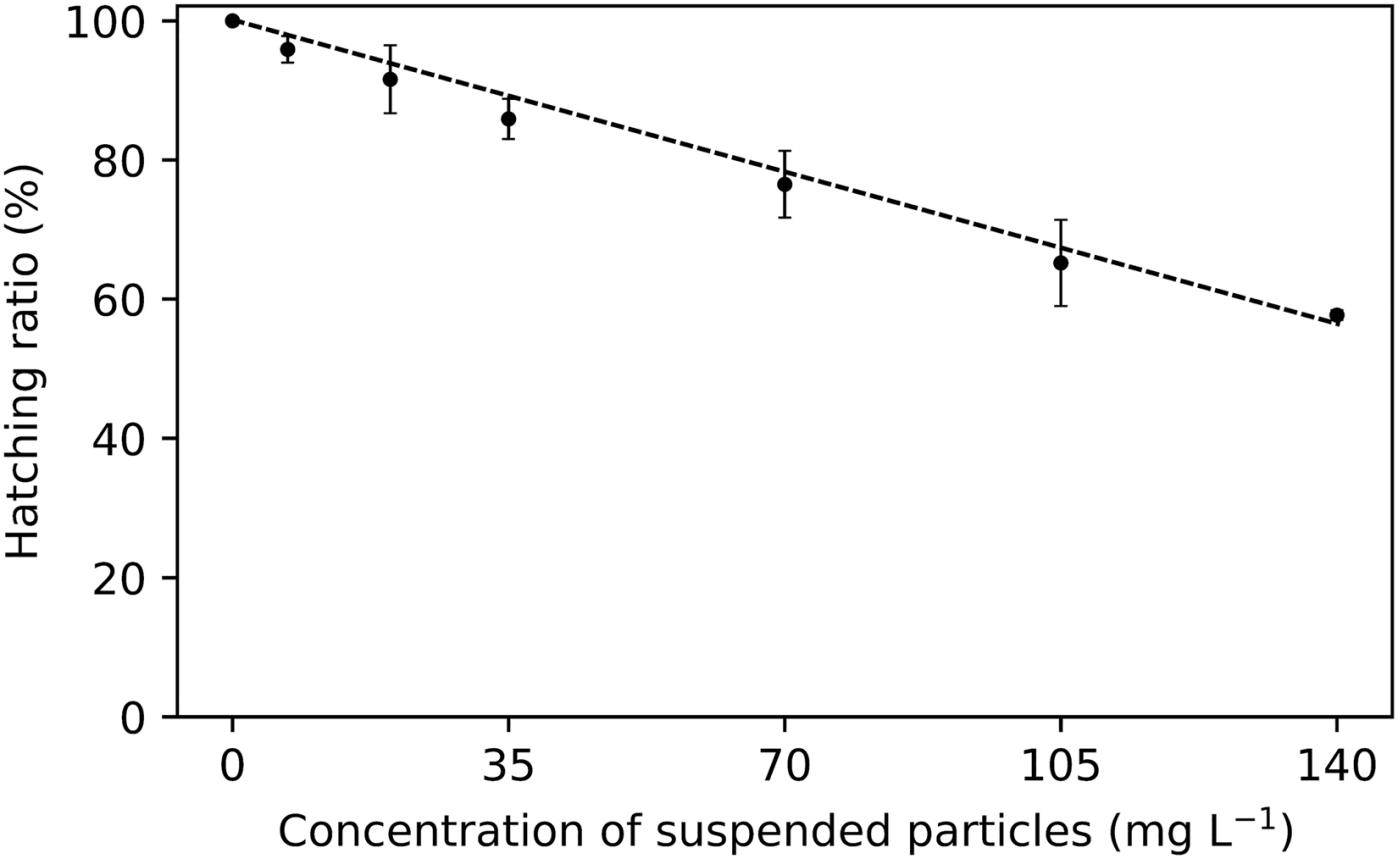
Relationship between suspended particle concentration and relative hatching ratio of pelagic shrimp *Lucensosergia lucens* eggs (*p* < 0.05).

The relative hatching ratio decreased linearly (Pearson’s correlation coefficient, *p* < 0.05) with increasing concentrations of suspended particles (Fig. 1). The lowest hatching ratio (57.7 ± 0.7%) was observed at the highest concentrations of suspended particles (139 mg L^−1^), while the hatching ratio at 35 and 70 mg L^−1^ were 85.9 ± 2.9% and 76.5 ± 4.8%, respectively. Furthermore, several abnormalities were observed in eggs and larvae after exposure to various concentrations of suspended particles, including deformed eggs, undeveloped body parts, and incomplete antennae (Supplementary Fig. S1). Effects of phenotypic variation on mortality were overlooked because it was beyond the scope of this study. Moreover, phenotypic variation is a common phenomenon within a local population and thus the effects observed are a representation of the population.

The relationship between the concentration of suspended particles and the relative hatching ratio was estimated as:

1 $$H_{r} = \, - 0.{32}C + { 1}00$$

where *H*_*r*_ and *C* are the relative hatching ratio and suspended particle concentration, respectively (*r*^2^ = 0.99, Fig. 1).

### Survival of nauplius larvae

**Figure 2:**
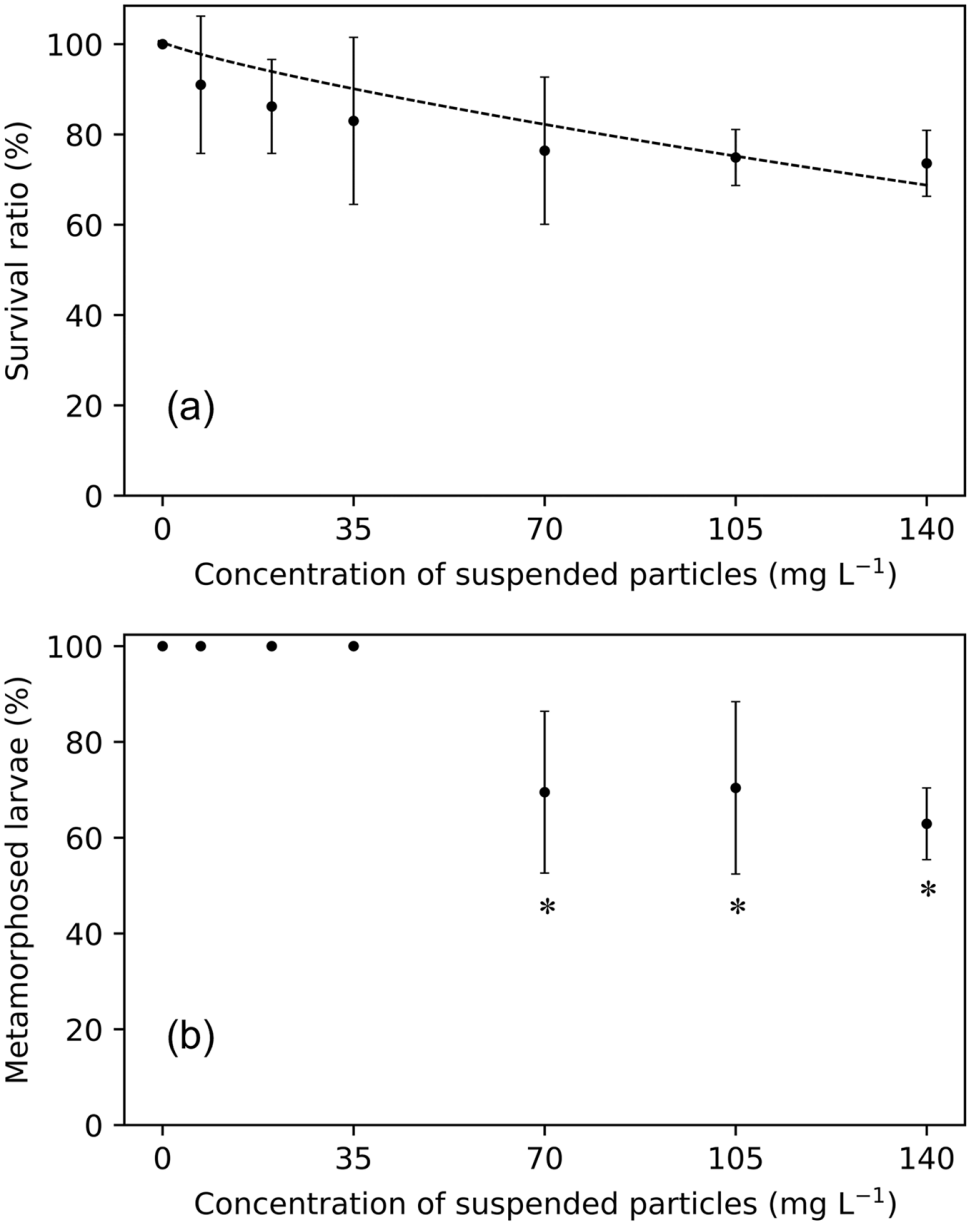
Survival and metamorphosis ratio of nauplius larvae; (**a**) relationship between relative survival ratio of nauplius larvae and suspended particle concentration (*p* < 0.05), and (**b**) metamorphosis ratio (%) from nauplius to elaphocaris larvae; after 72 h of exposure in different suspended particle concentrations. An asterisk (*) indicates statistical differences at (*p* < 0.05) when compared with the control condition.

Nauplius larvae hatched in filtered seawater were placed in different suspended particle concentrations and reared for 72 h. The survival of larvae was examined in all concentrations of suspended particles after 24, 48, and 72 h of exposure. The survival ratio (%) of larvae decreased with exposure time along with increasing suspended particle concentration (Table 1). No larval mortality was observed after 24 h in no kaolinite concentration, whereas the survival ratio was 91.7 ± 14.4% and 92.2 ± 7% at 35 mg L^−1^ and 139 mg L^−1^ of kaolinite particles, respectively. Increased temporal exposure to the same concentrations yielded increased larval mortality. After 72 h of exposure, the lowest survival ratio was recorded as 73.6 ± 7.3% at 139 mg L^−1^. Based on the exponential approximation the relationship between the concentration of suspended particles and relative survival ratio after 72 h of exposure was estimated as:

2 $$S_{n} = {1}00{\text{ exp }}\left( { - 0.00277C} \right)$$

where *S*_*n*_ and *C* are the relative survival ratio of nauplius larvae and suspended particle concentration, respectively (*r*^2^ = 0.87; Fig. 2a).

**Table 1:** Survival ratios (%) of nauplius larvae exposed to different suspended particle concentrations.

Concentration (mg L^−1^)	Survival ratio (%) of larvae at different time interval (hours)		
	24 h	48 h	72 h
No kaolinite	100 ± 0	100 ± 0	100 ± 0
6.9	100 ± 0	100 ± 0	91 ± 15.2
20	96.2 ± 6.4	96.2 ± 3	86.2 ± 10.4
35	91.7 ± 14.4	86.5 ± 28.8	83 ± 27.4
70	90.9 ± 3.2	82.8 ± 15.9	76.4 ± 21.3
104	89.1 ± 3.5	79 ± 13.1	74.9 ± 6.2
139	92.2 ± 7	75 ± 8.5	73.6 ± 7.3

All values are expressed as mean ± SD (*n* = 3).

The ratio of metamorphosis from nauplius to elaphocaris larvae decreased significantly as suspended particle concentration increased (*p* < 0.05). There was a complete metamorphosis from nauplius to elaphocaris larvae in low kaolinite concentrations (< 35 mg L^−1^); after that the metamorphosis sharply declined to 70% at 70 mg L^−1^ and 104 mg L^−1^. The lowest ratio (62.9%) was observed in larvae reared at 139 mg L^−1^ (Fig. 2b).

### Survival and growth of elaphocaris larvae

**Figure 3:**
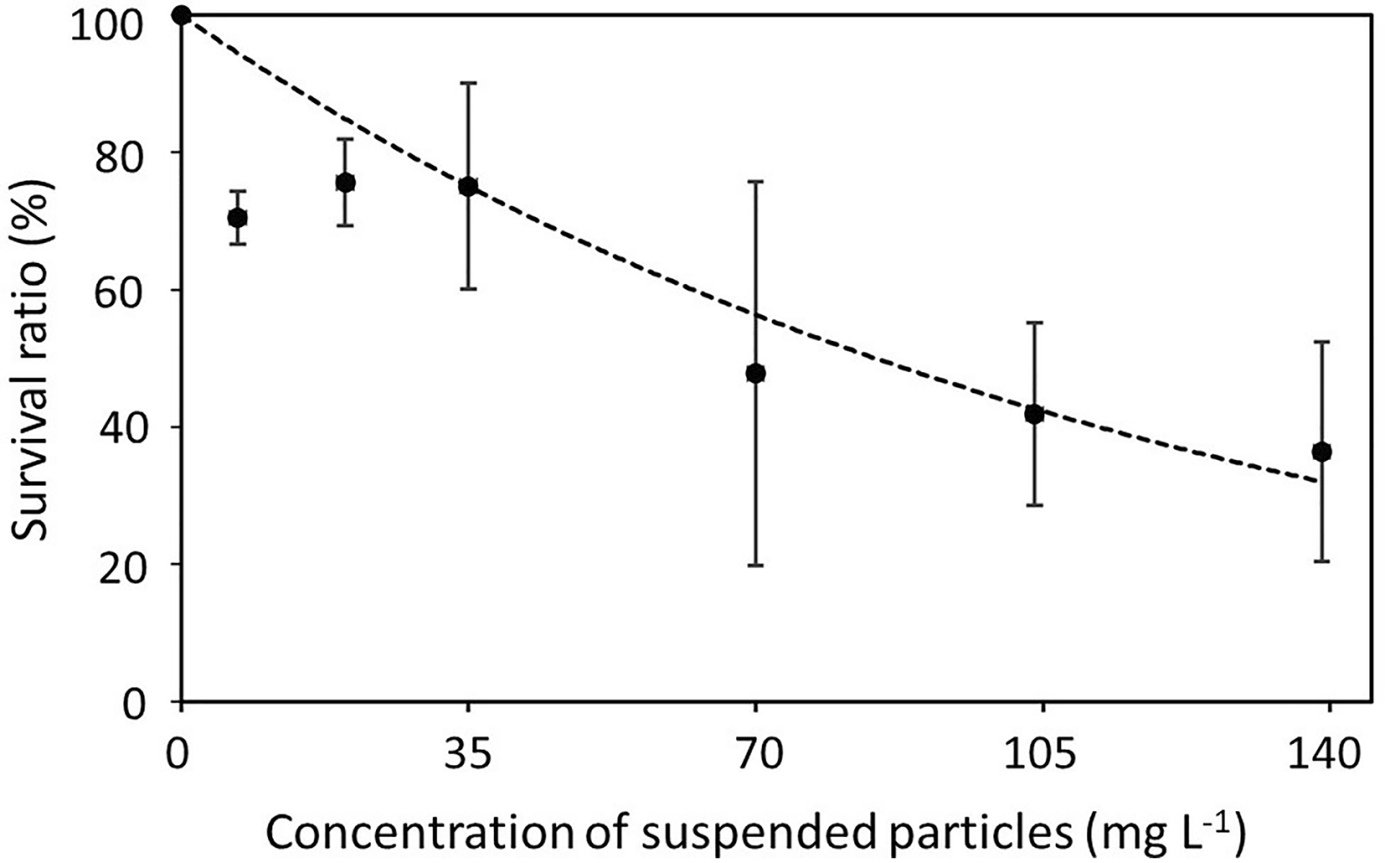
Relationship between suspended particle concentration and relative survival ratio of elaphocaris larvae after 8 days of exposure (*p* < 0.05).

The concentration of suspended particles significantly affected the survival ratio of elaphocaris larvae (*p* < 0.05; Table 2). In all seven concentrations, survival was greater than 80% on the third day of the experiment. After 6 days, survival ratios decreased sharply from their initial values. For example, in 6.9 mg L^−1^ of kaolinite, the survival was 93.3 ± 6.6% on the 6th day and fell to 70.5 ± 3.9% on the 8th day. In the absence of kaolinite, 100% of elaphocaris larvae had developed to elaphocaris II stage by the 8th day; however, delayed development was observed for all larvae when they were exposed to > 20 mg L^−1^ of kaolinite. Additionally, higher concentrations had stronger negative effects on survival. The highest survival was observed in 0 mg L^−1^ of kaolinite, while larvae in concentrations > 35 mg L^−1^ consistently exhibited low survival ratios throughout the experiment. On the 8^th^ day, the highest particulate concentration tested in this study (139 mg L^−1^) resulted in the lowest survival ratio of 36.4 ± 19.7%, compared with 47.8 ± 36.1% at 70 mg L^−1^, 75.1 ± 18% at 35 mg L^−1^, and 100 ± 0% at 0 mg L^−1^ of kaolinite particles. The exponential approximation of the relative survival ratio for different particle concentrations after 8 days of exposure gave the following relationship:

3 $$S_{e} = { 1}00{\text{ exp }}\left( { - 0.00819
C} \right)$$

where *S*_*e*_ and *C* are the relative survival ratio of elaphocaris larvae and suspended particle concentration, respectively (*r*^2^ = 0.82; Fig. 3).

**Table 2:** Survival ratios (%) of elaphocaris larvae exposed to different suspended particle concentrations.

Concentration (mg L^−1^)	Survival ratio (%) of larvae at different time interval (days)							
	Day 1	Day 2	Day 3	Day 4	Day 5	Day 6	Day 7	Day 8
No kaolinite	100 ± 0	100 ± 0	100 ± 0	100 ± 0	100 ± 0^c^	100 ± 0	100 ± 0^c^	100 ± 0^b^
6.9	97.7 ± 3.8	93.3 ± 6.6	93.3 ± 6.6	93.3 ± 6.6	93.3 ± 6.6^abc^	93.3 ± 6.6	88.8 ± 10.1^bc^	70.5 ± 3.9^ab^
20	97.7 ± 3.8	97.7 ± 3.8	97.7 ± 3.8	94.8 ± 8.8	94.8 ± 8.8^bc^	94.8 ± 8.8	88.9 ± 4.3^bc^	75.6 ± 6.3^ab^
35	100 ± 0	97.7 ± 3.8	95.5 ± 7.6	95.5 ± 7.6	95.5 ± 7.6^bc^	88.8 ± 10.1	84.4 ± 13.8^bc^	75.1 ± 18^ab^
70	100 ± 0	100 ± 0	95.5 ± 7.6	91.1 ± 15.3	82.2 ± 20.3^abc^	73.3 ± 30.5	64.4 ± 23.4^ab^	47.8 ± 36.1^a^
104	97.7 ± 3.8	93.4 ± 0.2	91.2 ± 3.9	80.2 ± 11.7	67.2 ± 7.5^a^	62.7 ± 11.1	49.8 ± 8.6^a^	41.9 ± 13.3^a^
139	97.7 ± 3.8	97.7 ± 3.8	84.4 ± 3.8	80 ± 6.6	71.1 ± 3.8^ab^	64.4 ± 7.6	48.8 ± 10.1^a^	36.4 ± 19.7^a^

Treatments with different superscripts are significantly different (*p* < 0.05). All values are expressed as means ± SD (*n* = 3).

**Figure 4:**
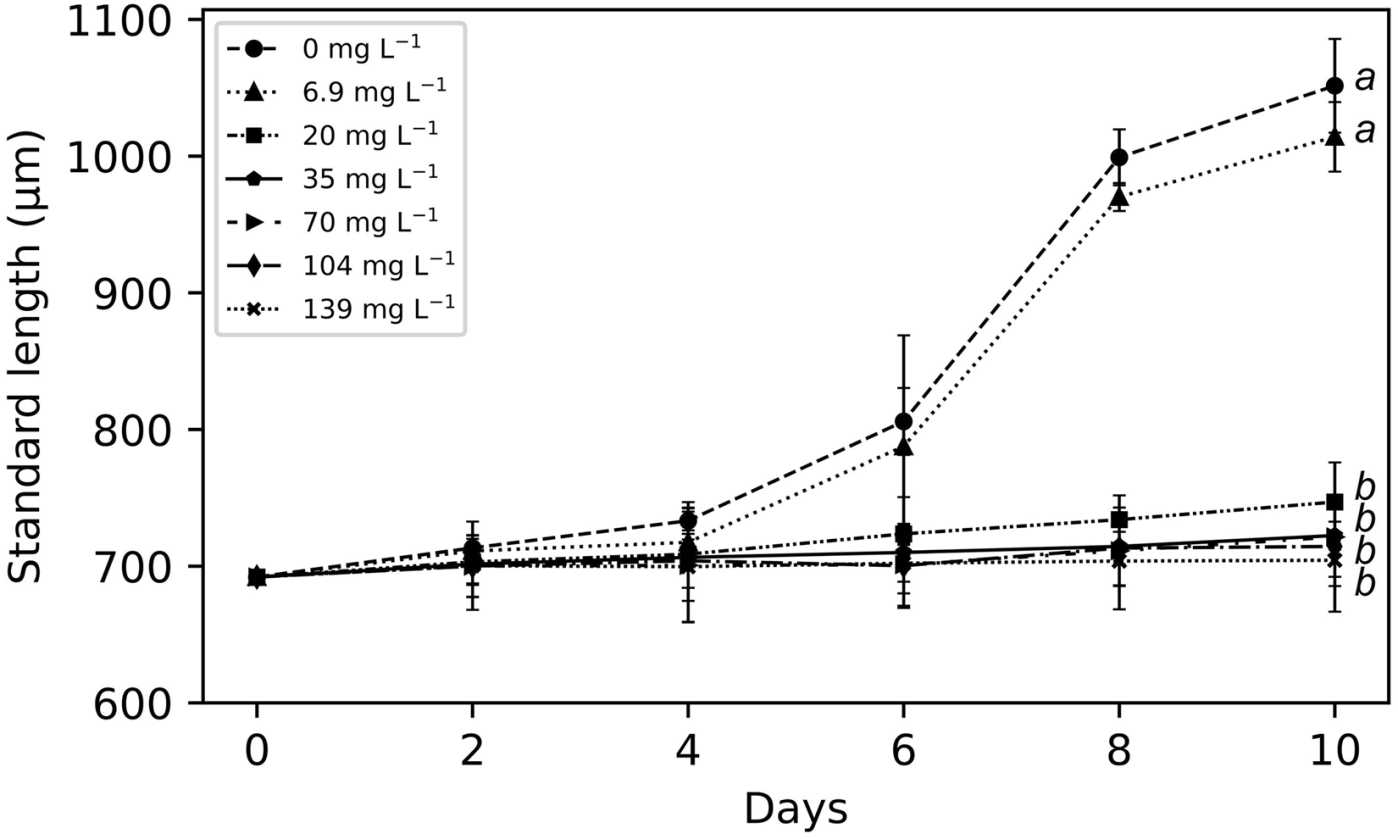
Growth comparison of elaphocaris larvae at different particle concentrations. Different letters at the right indicate significant differences (*p* < 0.05) among treatments after 10 days of exposure.

**Figure 5:**
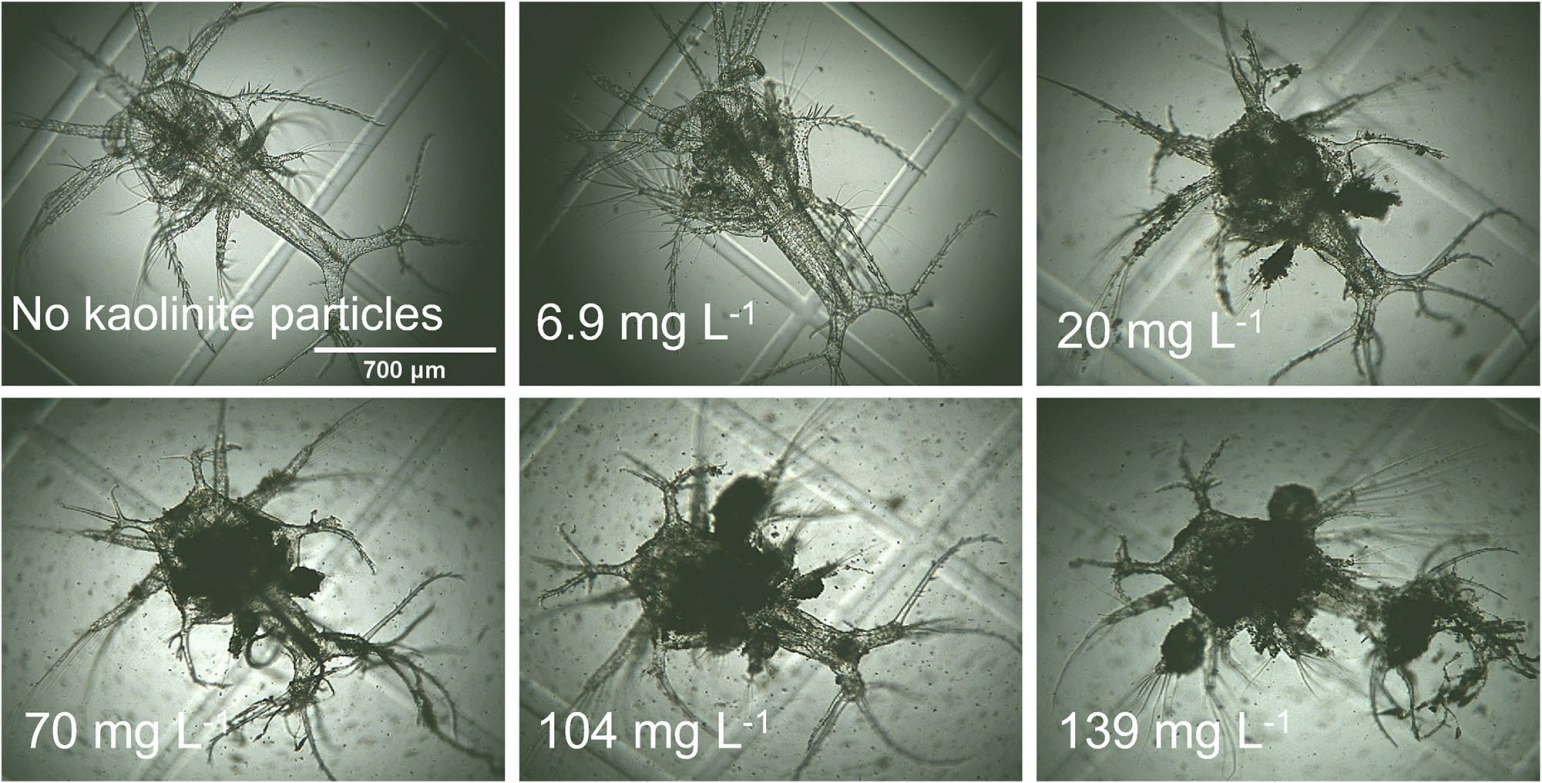
Appearance of elaphocaris larvae after 10 days of exposure to different suspended particle concentrations.

The mean standard length of elaphocaris larvae was 692 ± 0.6 µm at the start of the experiment. The growth of elaphocaris larvae at different particle concentrations is compared in Fig. 4. At low turbidity (0 and 6.9 mg L^−1^), larvae grew noticeably, but growth was lower at higher particle concentrations. Standard lengths recorded at 10 days were 1051.5 ± 34.3 µm, 1014.1 ± 25.4 µm, 746.9 ± 3.2 µm, 722.4 ± 10.2 µm, 721.3 ± 54.6 µm and 714.4 ± 29 µm at particle concentrations of 0 mg L^−1^, 6.9 mg L^−1^, 20 mg L^−1^, 35 mg L^−1^, 70 mg L^−1^, and 104 mg L^−1^, respectively. At the termination of the experiment, significant differences (*p* < 0.05) were found in the growth of larvae reared at different particle concentrations. Suspended particles readily adhered to the body surface of elaphocaris larvae, and the number of particles increased over time. Larvae were observed to be nearly covered with particles after 10 days of exposure to 139 mg L^−1^ (Fig. 5).

### Possible effect on early stages of *L. lucens* in Suruga Bay

The present study revealed the relationships between the concentration of suspended particles and the survival ratio of the early stage of *L. lucens.* In this study, we modeled the effects of suspended particles on egg hatching in Eq. (1), on nauplius larvae in Eq. (2), and on elaphocaris larvae in Eq. (3). By combining these equations, it is possible to quantitively determine the effects of suspended particles in seawater on *L. lucens* larvae from the egg stage to elaphocaris I larvae.

4 $$I_{L} = {1}00\left( {{1 } - H_{r} \times \, S_{n} \times \, S_{e} /{1}0^{{6}} } \right)$$

where, *I*_L_ indicates general loss of larvae (%) and *H*_*r*_*, S*_*n*_, and *S*_*e*_ denotes hatching success, nauplius larvae survival, and elaphocaris larvae survival, respectively.

This formula only calculates the effect of suspended particles on survival and does not take into account the possibility of delayed growth and development.

## Discussion

Our laboratory experiments indicated that inorganic particles have a great effect on the early life stages of *L. lucens.* In field observation, the mean turbidity at the spawning ground *of L. lucens* was recorded as 6.9 mg L^−1^ in the main spawning season (July–September). A higher portion of inorganic matter (67%) exists in seawater compared with the organic portion (33%). The maximum turbidity was recorded as 16 mg L^−1^ in the sea 13 km away from the estuary (unpublished). Substituting this value into Eqs. (4), the initial depletion of larvae was calculated to be 20%. However, this is a conservative value. Turbidity values from Fuji River revealed a much higher turbidity input suspended particles into Suruga Bay, especially after runoff during the rainy season (July–September), and mean values from 41.7 to 115.5 mg L^−1^ (http://www.pref.shizuoka.jp/kigyou/seibu/tousunn.html). If such high values continue for extended periods, they may affect the early stages of *L. lucens* from eggs to elaphocaris stages. With the rainy season corresponding to the hatching and early larval stages of *L. lucens*, the high inputs of suspended particles will have considerable effects on the population structure of *L. lucens* by affecting the hatching, metamorphosis, and growth of the larvae.

Despite inorganic suspended particles having a potential negative effect on the population, no study has examined the effects of inorganic particles on the *L. lucens* population. In natural oceanic environments, high turbidity is caused by the flow of particles from rivers and by the resuspension of the particles from the sea bottom during rough weather. When these processes continue for extended periods, they may inhibit the feeding and swimming of larvae. This might affect their survival and growth and, consequently, the recruitment process for *L. lucens.* The major spawning ground of *L. lucens* is in the area around the mouth of the Fuji River in Suruga Bay. This is also an area where muddy water flows in from the mouth of the Fuji River during heavy rains (http://www1.river.go.jp). Therefore, larvae of *L. lucens* in Suruga Bay could be greatly affected by inorganic suspended particles from the Fuji River.

Several studies have examined the influence of suspended particles on the hatchability of invertebrate eggs. In the absence of kaolinite (0 mg L^−1^), Manila clam *Ruditapes philippinarum* embryos exhibited 100% hatching success, which fell to 17% when exposed to 200 mg L^−1^ of suspended particles^15^. A significant decrease in hatchability was observed in American oysters *Crassostrea virginica* exposed to different silt concentrations^16^. Only 31% of eggs hatched when exposed to 500 mg L^−1^ of silt. A similar reduction in hatchability was reported for Venus clam *Mercenaria mercenaria* embryos exposed to 500 mg L^−1^ of kaolin and to 125 mg L^−1^ of Fuller’s earth^31^. The results of the present study suggested that *L. lucens* was more tolerant to suspended particles than *R. philippinarum*; i.e., hatchability of *L. lucens* eggs was higher than that of *R. philippinarum* at a similar concentration. However, *L. lucens* was less tolerant to particles than the *C. virginica* and *M. mercenaria*.

We demonstrated that the survivability of larvae decreased with increasing particle concentration and exposure time. Similar studies on other invertebrates found similar trends. Late juveniles of the kuruma shrimp *Marsupenaeus japonicus* exhibited 68% survival when exposed to 370 mg L^−1^ of particles after 21 days of culture^30^, higher than observed in this study. A survival rate of 60% was observed in the mysid shrimp *Mysidopsis bahia* exposed to 230 mg L^−1^ of suspended particles for 28 days^32^, whereas the current study recorded only a 36.4% relative survival ratio for elaphocaris larvae exposed to 139 mg L^−1^ of particles after 8 days of exposure. Suspended particles have also been implicated in the mortality of other crustaceans, including spot-tailed sand shrimp *Crangon nigromaculata* and grass shrimp *Palaemon macrodactylus*^14^, crab *Cancer magister*^14,33^, and black-tailed sand shrimp *Crangon nigricauda*^34,35^. Our data suggest that the effects of turbidity on the survival of *L. lucens* larvae are somewhat larger than for other shrimp. However, most earlier research focused on juvenile and post-larval stages, which may be less vulnerable to suspended particles.

Successful larval metamorphosis from nauplius to elaphocaris was significantly affected by turbidity (Fig. 2b). Delayed metamorphosis impaired survival and decreased the body size of crabs^36^ and shrimp^37^. Delayed metamorphosis accompanied by growth abnormalities are commonly observed in turbid conditions^38^. Delayed metamorphosis would also retard maturation to adulthood, which might also explain the observed population decline.

In the present study, we observed that *L. lucens* growth rates were significantly lower at high suspended particle concentrations. This effect has also been observed in filter-feeding invertebrates, e.g., juvenile *M. mercenaria*^39^, which showed inhibited larval growth at 23 mg L^−1^. In another study of that species, a suspended particle concentration of 44 mg L^−1^ retarded growth^40^. Kaolinite particle concentrations above 30 mg L^−1^ were found to inhibit the growth of *R. philippinarum* larvae^15^, whereas in the present study the growth of elaphocaris larvae was inhibited at concentrations above 20 mg L^−1^. Thus, the growth of *L. lucens*, *M. mercenaria*, and *R. philippinarum* was hampered by exposure to similar concentrations of suspended particles*.* Exposure to suspended inorganic particles also reduced growth rates of the larvae of American oyster *C. virginica*^16^, juvenile *Acanthochromis polyacanthus*^41^, adult surf clams *Spisula solidissima*^42^, hard clams *M. mercenaria*^43^, and soft-shell clams *Mya arenaria*^44^.

We observed that eggs that failed to hatch were often completely covered by particles. Similar effects of turbidity in marine environments on the hatching of fertilized eggs have been attributed to the adherence of large numbers of particles to the egg surface, which inhibits gas exchange. Gleason et al*.*^45^ noted that suspended particles adhering to eggs caused an oxygen shortage during embryonic development, which affected hatchability. In addition, suspended solids were believed to interfere with environmental cues that trigger hatching, e.g., temperature and light^46^.

In contrast, our study results indicated that the effects of kaolinite on nauplius and elaphocaris larvae were directly related to the attachment of particles to the body and gills, and to their ingestion. Adhesion of particles to the body surface results from sedimentation, which inhibits larval swimming behavior. Attachment of particles to the gills would interfere with respiratory gas exchange. We observed large numbers of kaolinite particles adhering to the body surface of *L. lucens*, particularly at higher turbidities (Fig. 5). Adhesion of particles to the body surface has been suggested as a cause of mortality in crustaceans^34,35^. Particles were reported to clog the gills, resulting in physiological stress and oxygen deprivation, ultimately leading to death^47,48^.

Based on their survival ratios after 3 days, nauplius larvae appear to be more susceptible to suspended particles than elaphocaris larvae. That is, the survival of nauplius larvae was lower than that of elaphocaris larvae after exposure to a similar concentration of kaolinite for the same duration. The greater sensitivity of early developmental stages may be related to their higher surface area to volume ratio resulting in the attachment of a relatively greater numbers of particles.

Metamorphosis is usually induced by biological, chemical and physical factors^38^. However, individuals may delay metamorphosis under unfavorable conditions, apparently prioritizing survival over development. Metamorphosis may resume when conditions become more favorable^38^.

Ingestion of particles by larvae could obstruct the digestive tract, which would interfere with the digestion and uptake of nutrients, reducing growth rate^15^. Very high intakes of suspended particles might cause starvation resulting in death^49^. In our study, the stomachs of elaphocaris larvae were discovered to contain suspended particles, which was indicative of ingestion. This would have affected their digestive functions and swimming behavior, resulting in lower growth and increased mortality.

This study made it possible for us to understand the initial decline in survival based on the particle amount, and in the future, it will be necessary to study the effects of sub-micro size inorganic particles. Continuous measurements of the turbidity levels in Suruga Bay should be carried out to better understand the extent of the effect of turbidity on this important shrimp resource.

## Methods

### Turbid seawater

The clay mineral kaolinite in powder form was used as inorganic suspended particles. Natural seawater with a salinity of 32 PSU was collected from the Pacific coast of Chiba Prefecture, Japan. The seawater was filtered through a 0.45 µm membrane filter (Merck, Darmstadt, Germany) and sterilized by autoclaving (model: SP 500, Yamato Co. Ltd., Tokyo, Japan). To create turbid water, weighed aliquots of kaolinite particles (20–6000 mg) were added to 3 L beakers containing 2 L of filtered seawater. The contents of the beaker were stirred for 10 min using a magnetic stirrer and allowed to stand for 10 min. Then, the supernatant was collected from the beaker and the suspended particle concentration was measured. The relationship between the mass of kaolinite added and the concentration of suspended particles in the supernatant is shown in Supplementary Fig. S2 and used to adjust the turbidity of the experimental media. The experimental turbidity condition ranged between 0 and 139 mg L^−1^, based on the assumption of natural field conditions. The mean diameter of suspended particles was 1.3 µm and ranged between 0.5 and 27.2 µm (Supplementary Fig. S3). Particle size was measured using a laser-diffraction particle-size analyzer (model: SALD-2300, Shimadzu Corporation, Tokyo, Japan).

### Spawning of brood shrimp and egg collection

Mature female pelagic shrimp *L. lucens* were collected by fishermen in the inner part of Suruga Bay (35°05′3.35″N, 138°34′11.2″E) in May 2021 and transported to the laboratory in temperature-controlled bags. The mean body length and wet weight of the shrimp were 47.4 ± 2.4 mm and 0.42 ± 0.04 g, respectively. Six brood shrimp were reared in separate 3 L beakers with sterilized filtered seawater in the dark to initiate spawning^2^. Temperature (20 ± 0.1 °C), salinity (31.8 ± 0.2 PSU), and dissolved oxygen (5.4 ± 0.03 mg L^−1^) were recorded and maintained within this optimum range during the rearing period. The shrimp were spawned in the morning of the day after collection.

The spawning was synchronized into two groups: Group A and Group B. Immediately after spawning, the fertilized eggs from each female within the synchronized groups were put together, i.e., beaker A (3 L) for Group A, and beaker B (3 L) for Group B. The eggs were retrieved from different females with different phenotypic variations; thus, the effects of kaolinite may be contingent upon a particular trait. However, this was not the focus of the current study. Group A was used in the analysis of the hatching ratio and Group B was cultured in beaker B in the controlled environment and allowed to grow to nauplius and elaphocaris larval stages. The Animal Welfare and Ethical Committee, Tokyo University of Marine Science and Technology, approved the experimental procedures used in this study.

### Egg hatching

**Figure 6:**
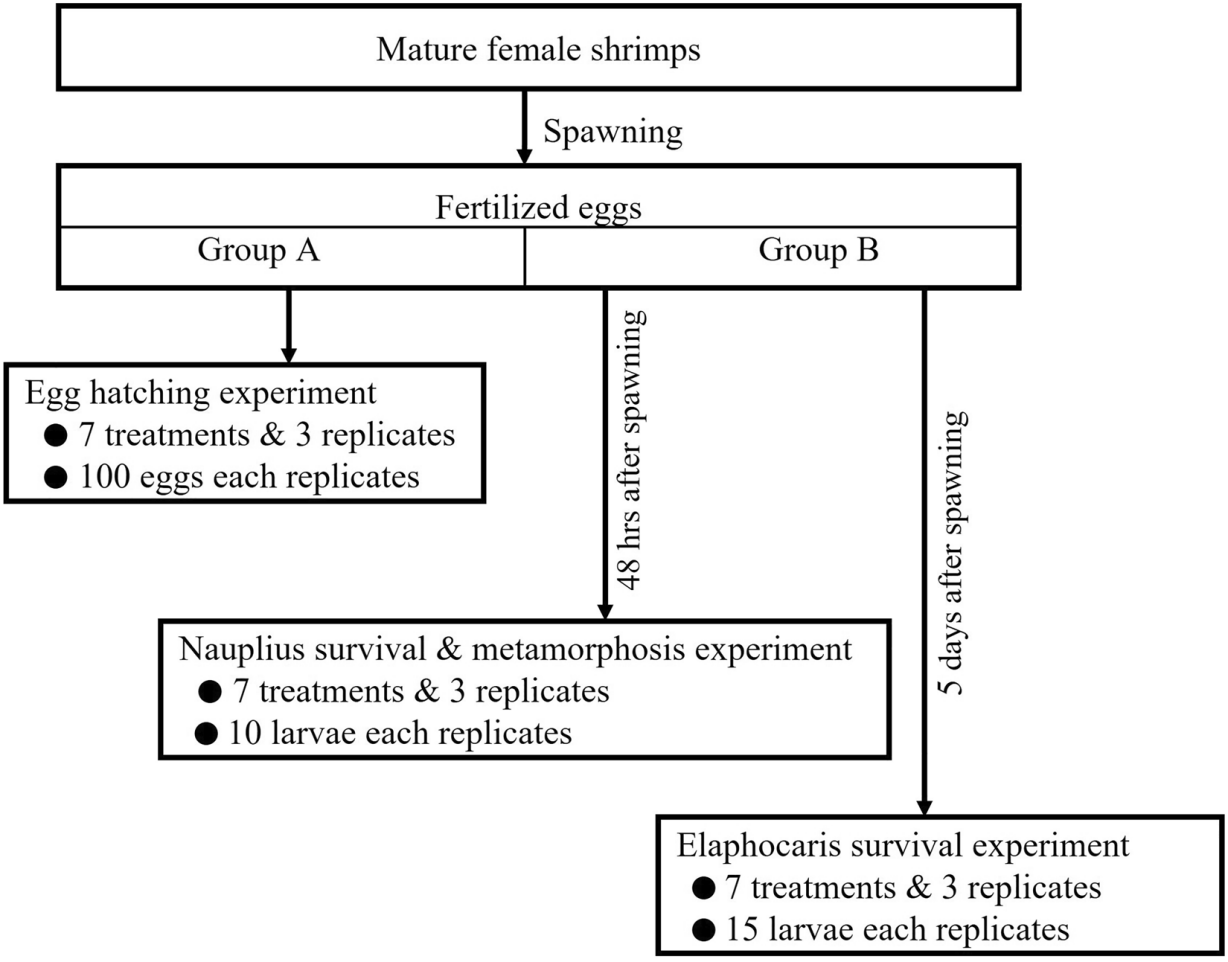
Schematic diagram of the experimental procedure in this study.

Immediately after spawning, fertilized eggs were randomly collected from the Group A beaker using a pipette and put in 50 mL tubes with a range of concentrations of suspended inorganic particles (no kaolinite, 6.9, 20, 35, 70, 104 and 139 mg L^−1^), with three replicates of each concentration. Each tube had 100 fertilized eggs. The tubes were attached to tube rotators (model: TR-350, AS ONE Corporation, Osaka, Japan) at 3 rpm to maintain uniform turbidity. A schematic diagram of the experimental procedure is shown in Fig. 6 and the experimental apparatus for the study can be found in Supplementary Fig. S4. Temperature was maintained at 20 ± 0.2 °C in a temperature-controlled water bath. Thirty-eight hours after spawning, the rotator was stopped, and the hatched larvae were immediately fixed in 1% formalin solution and observed under a microscope (model: BH-2, Olympus Co. Ltd, Tokyo, Japan) to determine the hatching ratio. The hatching ratio (%) was calculated as follows:

$${\text{Hatching ratio }}\left( \% \right) \, = \, \left( {{\text{hatched egg number}}/{\text{total egg number}}} \right) \, \times { 1}00$$

The relative hatching ratio at each particle concentration was calculated by assuming the hatching ratio without kaolinite was 100%.

### Nauplius survival and metamorphosis

Nauplius larvae were obtained 48 h after spawning from the Group B beaker and placed into tubes using a pipette with a similar range of particle concentrations as used for the eggs. Each concentration was conducted in triplicate with 10 nauplius larvae in each replicate. The nauplius larvae were then cultured for 72 h, during which they metamorphosed into elaphocaris larvae. Survival rates were recorded at 24, 48, and 72 h from the onset of exposure. Nauplius larvae were observed and photographed under a microscope, and dead larvae were removed. The survival ratio (%) of nauplius larvae was calculated as follows:

$${\text{Survival ratio }}\left( \% \right) = \left( {{\text{number of surviving larvae}}/{\text{total number of larvae}}} \right) \times {1}00$$

The relative survival ratio at each particle concentration was calculated by assuming that the nauplius survival ratio without kaolinite particles was 100%.

After 72 h, the photographs were used to determine whether larvae had metamorphosed. Metamorphosis from nauplius to elaphocaris larvae was judged by their body transformation as described by Omori^2^. The metamorphosis ratio (%) was calculated as follows:

$${\text{Metamorphosis ratio }}\left( \% \right) \, = \, \left( {{\text{number of metamorphosed larvae}}/{\text{total number of larvae}}} \right) \, \times { 1}00$$

### Elaphocaris survival

Elaphocaris larvae were obtained 5 days after spawning from the Group B beaker and placed into tubes with a range of kaolinite concentrations as used in the above experiments. Each experiment was conducted in triplicate with 15 elaphocaris larvae in each replicate. The elaphocaris larvae were cultured for up to 10 days. Survival was checked every 24 h until the elaphocaris II stage, and dead larvae were discarded. Concentrations of suspended particles were renewed daily and *Chaetoceros gracilis* diatoms, cultured in an incubator, were supplied as food at a concentration of 20 × 10^3^ cells mL^−1^^50^. The survival ratio (%) of elaphocaris larvae was calculated as follows:

$${\text{Survival ratio }}\left( \% \right) \, = \, \left( {{\text{number of surviving larvae}}/{\text{total number of larvae}}} \right) \, \times { 1}00$$

The relative survival ratio at each particle concentration was calculated by assuming that the elaphocaris survival ratio without kaolinite particles was 100%.

Five larvae from each turbidity concentration were randomly sampled each day for microscopic observation and photographed to measure standard length (in dorsal view along the mid-sagittal plane from the anterior margin of the forehead to the posterior margin of the telson). Survival was judged from the presence or absence of movement under a microscope, particularly swimming and antennae motion after stimulation by touch. Death was also identified through discoloration of the larvae and settling at the bottom. The standard length of the body was measured using ImageJ software.

### Data analysis

The hatching and survival of larvae were presented as mean ± standard deviation (SD). Data were analyzed using one-way analysis of variance (ANOVA) followed by Tukey’s post hoc test to evaluate the statistical significance of differences among the different treatments. Statistical significance was set at the *p* < 0.05 level and analysis was performed using SPSS Version 16.0 for Windows (SPSS Inc., Chicago, IL).

## Supplementary Information

Supplementary Figures.

## Data Availability

This dataset is not publicly available, but data sharing will be considered upon request. Request for data sharing can be sent to the corresponding author.
