# Author Correction: Influence of suspended inorganic particles (kaolinite) on eggs and larvae of the pelagic shrimp *Lucensosergia lucens*

**DOI:** 10.1038/s41598-022-26015-2

**Published:** 2022-12-15

**Authors:** Md. Jahangir Alam, Kazuma Date, Hisayuki Arakawa

**Affiliations:** Tokyo University of Marine Science and Technology, 5‑7, Knan‑4, Minato, Tokyo, 108‑8477 Japan

Correction to: *Scientific Reports* 10.1038/s41598-022-18373-8, published online 18 August 2022

The original version of this Article contained an error in Equation 2 and Equation 3, where the values were interchanged.

2 $$S_{n} \, = \,100{\text{ exp }}( - 0.008C)$$

3 $$S_{e} \, = \,100{\text{ exp }}( - 0.003C)$$

now reads:

2 $$S_{n} \, = \,100{\text{ exp }}( -{0.00} {\bf{277}}C)$$

3 $$S_{e} \, = \,100{\text{ exp }}( -{0.00} {\mathbf{819}}C)$$

The original Article has been corrected.

