# Supplementary material for: Influence of suspended inorganic particles (kaolinite) on eggs and larvae of the pelagic shrimp Lucensosergia lucens
Source: Sci Rep. 2022 Aug 18;12:14085. doi: 10.1038/s41598-022-18373-8 (PMC9388537; doi:10.1038/s41598-022-18373-8)
Supplement: Supplementary file 1 — Supplementary Figures. [file 41598_2022_18373_MOESM1_ESM.pdf]

Influence of suspended inorganic particles (kaolinite) on eggs and larvae of the pelagic shrimp *Lucensosergia lucens*

Authors: Md. Jahangir Alam*, Kazuma Date, Hisayuki Arakawa

Affiliation: Tokyo University of Marine Science and Technology, 5-7, Konan-4, Minato, 108-8477, Tokyo, Japan

*Corresponding author: jahangirfmn@pstu.ac.bd

Supplementary information

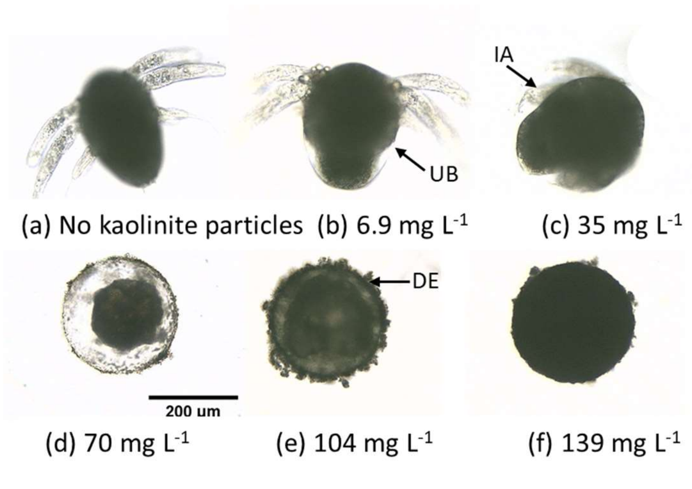

Figure S1. Morphological deformities observed in after 38 hours from spawning induced by different suspended particle concentrations; (a) normal hatched larvae, (b) undeveloped body parts (UB), (c) incomplete antennae (IA), (d) unhatched embryo, (e) deformed egg (DE), and (f) egg covered by particles.

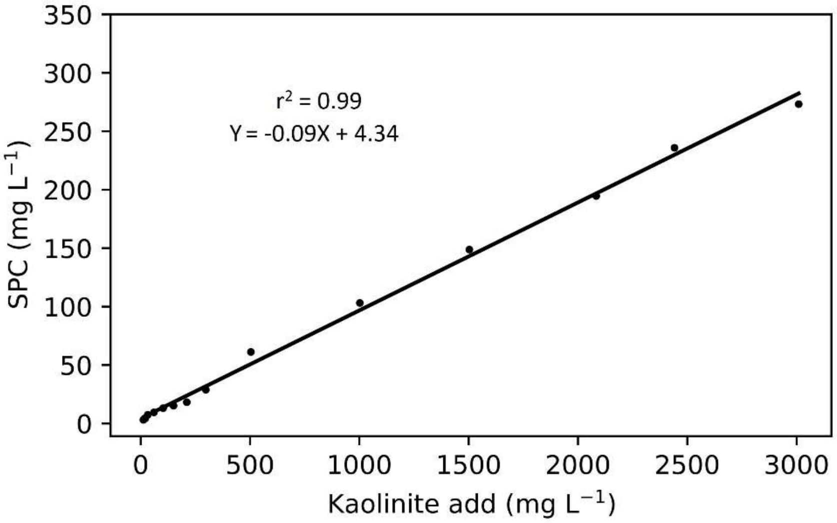

Figure S2. Correlation between the amount of kaolinite add and suspended particle concentration (SPC) of the supernatant.

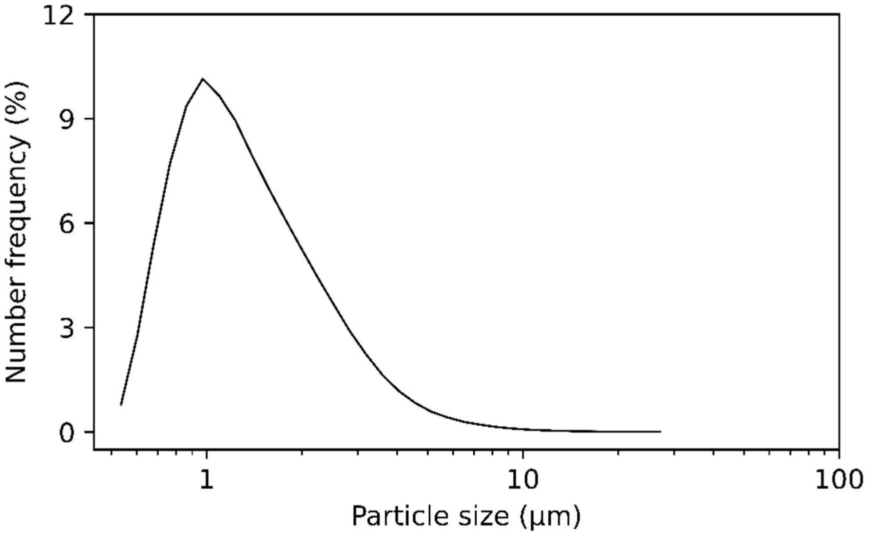

Figure S3. Particle size distribution of kaolinite

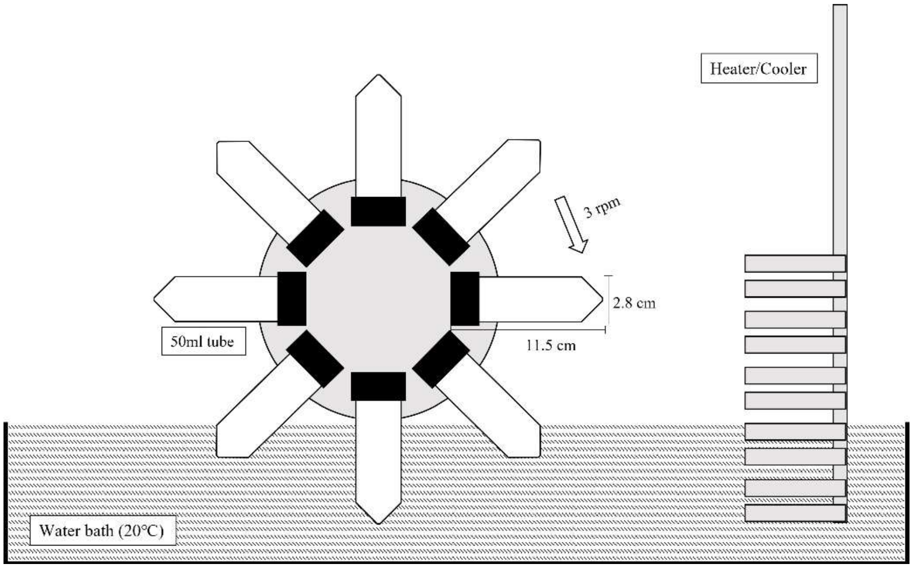

Figure S4. Experimental apparatus for this experiment.
